# Expression, purification and characterization of *Mycobacterium tuberculosis* RpfE protein^☆^

**DOI:** 10.1016/S1674-8301(12)60003-7

**Published:** 2012-01

**Authors:** Ying Xue, Yinlan Bai, Xue Gao, Hong Jiang, Limei Wang, Hui Gao, Zhikai Xu

**Affiliations:** a Department of Radiation Therapy, Xijing Hospital, the Fourth Military Medical University, Xian, Shaanxi 710032, China;; b Department of Microbiology, School of Basic Medicine, the Fourth Military Medical University, Xian, Shaanxi 710032, China.

**Keywords:** resuscitation-promoting factor (RpfE), purification, *Mycobacterium tuberculosis*, *Mycobacterium vaccae*

## Abstract

Resuscitation promoting factor E (RpfE) is one of the five Rpf-like proteins in *Mycobacterium tuberculosis (M. tuberculosis)*. These Rpf-like proteins are secretory, which make them candidates for recognition by the host immune system. In this study, the *RpfE* gene was amplified from *M. tuberculosis*, cloned into the expression vectors pDE22 and pPRO EXHT, and were expressed in *Mycobacterium vaccae (M. vaccae)* and *Escherichia coli* DH5α, respectively. Both recombinant RpfE proteins were purified by Ni-Sepharose affinity chromatography, and were given to C57BL/6 mice. The RpfE proteins elicited T cell proliferation, and stimulated the production of gamma interferon (IFN-γ), interleukin-10 (IL-10) and IL-12. Our results indicated that the RpfE protein expressed in *M. vaccae* could more efficiently stimulate cellular immune response, making it a promising candidate as a subunit vaccine.

## INTRODUCTION

Tuberculosis (TB) is a chronic infectious disease caused by the pathogen *Mycobacterium tuberculosis* (*M. tuberculosis*)[1]. The World Health Organization (WHO) reported that there were an estimated 8.8 million new TB cases in 2005, and 1.6 million people died of the disease, including 195,000 patients infected with HIV (WHO Report 2007, http://www.who.int/tb/en/). The current epidemic mainly results from the lack of an efficient vaccine, development of drug resistance in the pathogen, and deadly synergy of coinfection with HIV[2].

*Mycobacterium bovis (M. bovis)* bacillus Calmette-Guérin (BCG), an attenuated strain of *M. bovis*, has been the only accepted vaccine for the prevention of TB for decades[3],[4]. Although the current vaccine is effective for protection against childhood forms of TB[5], it has failed to prevent adult pulmonary manifestations of the disease in countries where TB is highly endemic[6]. Moreover, BCG immunization is contraindicated for HIV-infected persons since inoculation of BCG may cause life-threatening diseases in immunocompromised individuals[7],[8]. Since 1997, over 170 vaccine candidates have been tested using mice and guinea-pigs in low-dose, aerosol challenge models of primary TB[9]. Recombinant BCG strains, DNA-based vaccines, live attenuated *M. tuberculosis* vaccines and subunit vaccines formulated with novel adjuvants have shown promise in preclinical animal challenge models[10]. Three of these vaccines are being evaluated at present in human clinical studies, and several other vaccine preparations are being targeted for clinical trials in the near future[11]. Therefore, development of new or better vaccines is urgently needed to counter the global threat of the disease.

*M. luteus* secretes a small protein called resuscitation-promoting factor (Rpf), which has autocrine and paracrine signaling functions and is required for the resuscitation of dormant cells[12]. Rpf can increase the viable cell count of dormant *M. luteus* cultures at least 100-fold and can also stimulate the growth of viable cells[13],[14]. Similar genes are widely distributed among high G + C Gram-positive bacteria, and genome sequencing has uncovered examples in *M. leprae, M. tuberculosis, M. bovis, Streptomyces spp*. and *Corynebacterium glutamicum*[13]. *M. tuberculosis* possesses five genes with significant homology to the *Rpf* of *M. luteus*. *RpfA* (Rv0867c), *RpfB* (Rv1009), *RpfC* (Rv1884c), *RpfD* (Rv2389c) and *RpfE* (Rv2450c) share a conserved segment, which encodes an Rpf-like domain of about 70 residues long[15]. More recently, the Rpf-like proteins of *M. tuberculosis* have been shown to stimulate the growth of extended-stationary-phase cultures of *M. bovis* BCG[12]. Our previous study also showed that purified recombinant RpfD could stimulate the resuscitation of *M. tuberculosis* H37Ra[16]. These data suggest that the Rpf proteins can influence the growth of mycobacteria[17]. Surprisingly, all of the five individual *rpf* deletion mutant strains showed growth kinetics similar to the wildtype strain, likely due to the redundancy[15],[18]. Bacteria with deletion of multiple *rpf* genes (such as *rpfA*-*C*-*B*, *rpfA*-*C*-*D*) were unable to resuscitate, demonstrating the importance of the Rpf-like proteins of *M. tuberculosis* in resuscitation from the nonculturable state[18]. Sequence analysis suggests that at least some of these proteins are secreted and that all five proteins probably have extracytoplasmic functions[19], making them potential targets for recognition by the host immune system at the stage of reactivated disease. Therefore, these proteins have potential as novel diagnostic reagents and subunit vaccine candidates for control of TB. In this study, we described the expression and purification of recombinant RpfE proteins in *E. coli* (iRpfE) and *M. vaccae* (sRpfE) with regard to their immunogenic properties.

## MATERIALS AND METHODS

### Bacterial strains, plasmids and animals

*M. tuberculosis* H37Rv and *M. bovis* BCG were grown in Middlebrook 7H9 medium supplemented with 0.2% glycerol, 0.05% Tween 80 and 10% oleic albumin dextrose catalase (OADC) enrichment (Becton Dickinson, NJ, USA) at 37°C. The bacteria were grown to an optical density at 600 nm of 1 in roller bottles, divided into 1 mL aliquots in cryovials, and stored at -70°C. *E. coli* DH5α and *M. vaccae* were grown on solid or in liquid Luria-Bertani medium. The expression vectors pPRO-EXHT (Invitrogen Life technologies, USA) and pDE22 (a shuttle secretory plasmid for *M. smegmatis*, our unpublished data) were used for protein expression. C57BL/6 mice were bred under conventional conditions in the animal facility of the Animal Center of the Fourth Military Medical University. Female mice, 8-10 w of age at the beginning of the experiment, were used. The study protocol was approved by the local institutional review board and all animal experiments were carried out in strict accordance with the established guidelines regarding animal use and care at the Fourth Military Medical University, Xian, China.

### Cloning of *rpfE* into expression vectors

Genomic DNA was isolated from *M. tuberculosis* H37Rv using a standard phenol/chloroform extraction protocol[20]. The *rpfE* gene was amplified from genomic DNA with a pair of primers which were designed based on the known *rpfE* DNA sequence (Tuberculist Accession No. Rv2450): 5′-CCGGGATCCCATCACCATCACCATCACATGAAGAACGCCCGTACGACG-3′, which contained an *Bam*H I site (underlined) and 18 residues encoding His-tag (double-underlined); and 5′-CCGAAGCTTTGCGTCTTTTCGCGGTGG-3′, which contained an *Hind* III site (underlined). The reactions were performed using r*Taq* polymerase (Takara, Dalian, China) in a final volume of 25 µL. The thermal cycling program was performed in a thermo cycler (MJ Research, Watertown, MA, USA) and the conditions were as follows: 30 cycles of 30 sec at 94°C, 30 sec at 58°C, and 60 sec at 72°C. The amplified product was digested with *BamH* I and *Hind* III, and then ligated to the corresponding sites of the expression vectors pPRO-EXHT and pDE22. Finally, both recombinant vectors were checked for the correct orientation and DNA sequence by sequencing in both directions (Invitrogen Life technologies, Beijing, China). The correct plasmids were designated as pPRO-EXHT-rpfE and pDE22-rpfE, respectively.

### Transformation of *E. coli* DH5α and *M. vaccae*

The competent cells of *E. coli* DH5α and *M. vaccae* were prepared as previously described[16]. For electroporation, 1-2 µL of pPRO-EXHT-rpfE and pDE22-rpfE plasmids were added to 0.4 mL of the competent *E. coli* DH5α and *M. vaccae* suspensions, respectively. The mixture was incubated on ice for 10 min and transferred into a 0.2 cm electrode gap electroporation cuvette (Bio-Rad, Hercules, CA, USA) and was subjected to a single-pulse electroporation of 25 µF at 2.5 kV, with resistance set at 1,000 Ω. After electrotransformation, the cuvettes were put back on ice for 10 min, and then the mixtures were transferred into 5 mL of LB broth. The culture was then incubated at 37°C for 2 h followed by centrifugation at 3,000 *g* for 10 min. *E. coli* DH5α cells were plated on LB agar plate containing 100 µg/mL ampicillin, and *M. vaccae* cells were plated on LB agar plate containing 100 µg/mL hygromycin. The plates were incubated at 37°C until colonies became visible.

### Expression and purification of recombinant iRpfE in *E. coli* DH5α

*E. coli* DH5α (pPRO-EXHT-rpfE) cells were grown in 200 mL of LB medium with shaking (100 *g*) at 37°C. When the culture reached an OD_600_ of 0.6, 1 mmol/L isopropyl-beta-D-thiogalactopyranoside (IPTG) was added to the culture. After induction for 4 h, the culture was centrifuged at 8,000 *g* for 10 min to harvest the cells. The degree of the expression was evaluated by 15% sodium dodecyl sulfate polyacrylamide gel electrophoresis (SDS-PAGE). The cells were then resuspended in 5 mL of lysis buffer (6 mol/L guanidine hydrochloride; 20 mmol/L sodium phosphate; 500 mmol/L NaCl; pH 7.8) and incubated at 25°C for 10 min before further disruption by sonication. In the process of cell lysis, 15 mmol/L protease inhibitor phenylmethanesulfonyl fluoride (PMSF) was added. Following sonication, centrifugation was performed to remove the insoluble cell debris and the supernatant was transferred into 4 mL of prepared Ni-sepharose for binding. After binding for 30 min, the insoluble recombinant iRpfE was eluted by 1 mL of washing buffer (8 mol/L urea; 20 mmol/L sodium phosphate; 500 mmol/L NaCl; pH 6.0) four times, 1 mL of washing buffer (8 mol/L urea; 20 mmol/L sodium phosphate; 500 mmol/L NaCl; pH 5.3) four times, and 1 mL of elution buffer (8 mol/L urea; 20 mmol/L sodium phosphate; 500 mmol/L NaCl; pH 4.0) four times. The production of purified protein was detected using 15% SDS-PAGE and the iRpfE was identified with anti Histag antibody using Western blotting analysis. The degree of purification was evaluated by calculating OD_280_ (Cecil Instruments Ltd., Cambridge, England). The purified iRpfE was refolded by dialyzing in 1 L of 6 mol/L urea for 4 h, 1 L of 4 mol/L urea for 4 h, 1 L of 2 mol/L urea for 4 h, 1 L of 1 mol/L urea for 4 h, and 1 L of 0.01 mol/L PBS for 4 h. The concentration of the refolded protein was determined by calculating OD_280_.

### Expression and purification of sRpfE in *M. vaccae*

Recombinant *M. vaccae* colonies were inoculated into 200 mL of LB medium and the culture was shaken at 37°C (100 *g*) until the OD_600_ value was reached. Then the culture was centrifuged at 8,000 *g* for 10 min and the supernatant was transferred into 10 tubes containing 2 mL of Ni-Sepharose that had been washed by native binding buffer (containing 50 mmol/L NaH_2_PO_4_, 0.5 mol/L NaCl and 10 mmol/L imidazole; pH 8.0) for binding. The binding was processed at 4°C for 60 min with gentle shaking. After binding for 60 min, the sepharose in each tube was pelleted at 800 *g* for 2 min, and collected together into a fresh tube. The sRpfE was then eluted with 1 mL of native washing buffer (containing 50 mmol/L NaH_2_PO_4_, 0.5 mol/L NaCl and 20 mmol/L imidazole; pH 8.0) five times. The protein was eluted with 1 mL of native elution buffer (containing 50 mmol/L NaH_2_PO_4_, 0.5 mol/L NaC and 250 mmol/L imidazole; pH 8.0) five times. The degree of purification was evaluated by 15% SDS-PAGE and the sRpfE was identified by Western blotting analysis using the anti His-tag antibody.

### Immunization of mice

Mice in the protein immunization group were injected subcutaneously with 0.1 mL of PBS mixed with 0.1 mL of incomplete Freund's adjuvant (IFA, Sigma) containing 10 µg iRpfE and sRpfE proteins, respectively. Mice in the BCG group were intravenously immunized with 3×10^7^ BCG in the tail vein. Animals were boosted twice at 2-week intervals. Control mice were injected with PBS mixed with IFA.

### Antibody responses

Immunized and control mice were sacrificed to obtain sera on d 21 after the last injection. Antibody responses were measured by enzyme-linked immunosorbent assay (ELISA). Microtiter plates were coated overnight in 0.1 mol/L carbonate buffer (pH 9.6) containing 10 µg/mL of iRpfE or sRpfE. Plates were blocked with 1% bovine serum albumin (BSA) in PBS at 37°C for 1 h. After washing, the sera samples were added with appropriate dilutions in 1% BSA and were incubated at 37°C for 1 h. The plates were then incubated with HRP-conjugated goat anti-mouse immunoglobulin G_1_ (IgG_1_) or IgG_2a_ monoclonal antibodies (Pharmingen) at 37°C for 1 h, and finally added with *p*-nitrophenylphosphate as substrate. The absorbance at 420 nm was measured.

### Proliferative response of T cells

Briefly, mouse splenocytes were seeded at 5×10^5^ cells per well in a 96-well plate containing 2 µg PPD per well, and were incubated at 37°C with 5% CO_2_ for 72 h. The plates were then added with 20 µL of MTT (5 mg/mL, diluted with PBS, pH 7.2) and incubated for 4 h. The supernatant of each well was then replaced with 150 µL DMSO. After 10 min of incubation, the absorbance of each well at 490 nm was measured. All cultures were performed in triplicate, and wells without stimulation of PPD served as controls. Stimulation index (SI) was calculated.

### Cytokine production

A total of 5×10^6^ splenocytes per well were cultured in 24-well plates. After 48 h of incubation, the supernatants from each well were harvested and stored at -20°C until used for testing. Interleukin-12 (IL-12), IL-10, and interferon gamma (IFN-γ) in the culture supernatants were detected using ELISA kits (Jingmei Company, China). Standard curves were generated with known concentrations of recombinant rIL-12, rIL-10 and rIFN-γ from the kits.

### Statistical analysis

One-way analysis of variance (ANOVA) followed by (Student-Newman-Keuls) SNK method, or repeated-measures ANOVA was performed using SPSS Version 17.0 for Windows (SPSS Inc., Chicago, Illinois, USA) to determine the statistical significance of differences between the immunization with different antigens. A *P*-value < 0.05 was considered statistically significant.

## RESULTS

### Cloning of the *rpfE* gene

*M. tuberculosis rpfE* was amplified from the genome of *M. tuberculosis* strain H37Rv by PCR using specific primers. The amplicon was then inserted into the cloning vector pGEM-T-Easy and verified by sequencing. The *rpfE* was subcloned into the expression vectors pPRO-EXHT and pDE22, and the recombinant plasmids were designated as pPRO-EXHT-rpfE and pDE22-rpfE, respectively.

### Expression and purification of recombinant iRpfE in *E. coli* DH5α

**Fig. 1:**
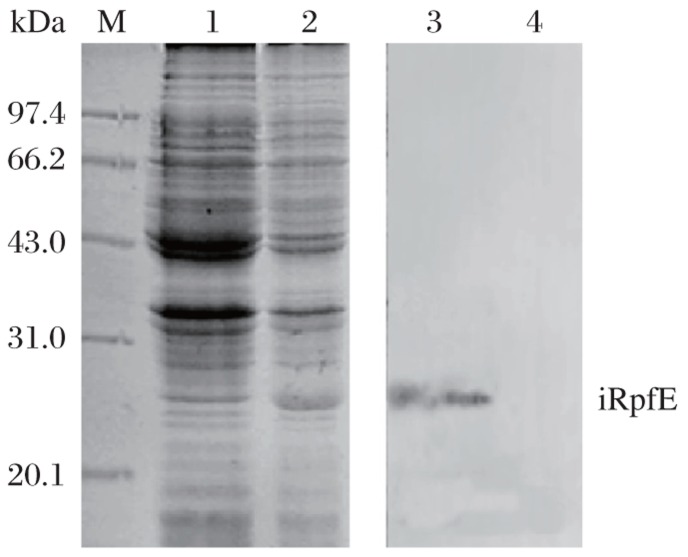
SDS-PAGE and Western blotting analysis of iRpfE. Lane M: molecular weight marker; Lane 1: uninduced E. coli DH5α (pPRO EXHT-rpfE); Lane 2: induced E. coli DH5α (pPRO EXHT-rpfE); lanes 3 and 4: Western blot analysis of induced (Lane 3) and uninduced (Lane 4) bacterial lysate using anti-His6 antibody.

**Fig. 2:**
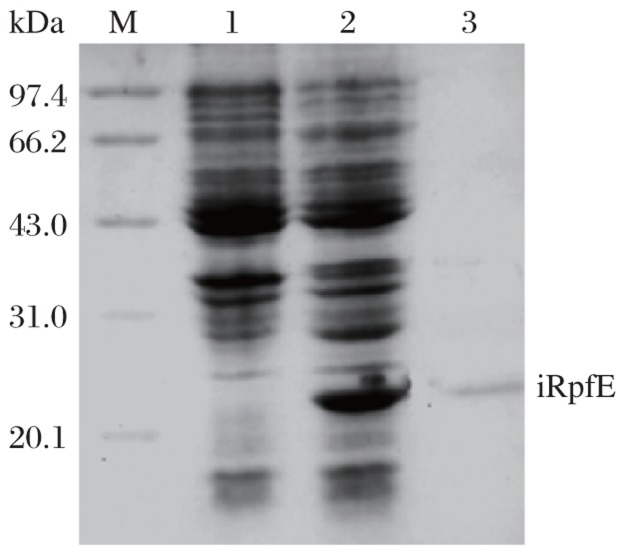
Analysis of purified sRpfE on 15% SDS-PAGE gel. Lane M: molecular weight marker; Lane 1: uninduced *E. coli* DH5α (pPRO EXHT-RpfE); Lane 2: induced *E. coli* DH5α (pPRO EXHT-RpfE); Lane 3: purified iRpfE protein after refolding.

Induction of E. coli DH5α (pPRO-EXHT-rpfE) resulted in a high-level expression of iRpfE, which accounted for approximately 16% of total cellular protein. The apparent molecular weight of this protein was about 22 kDa on SDS-PAGE, which is consistent with the estimated molecular weight of His-tagged RpfE. Western blot analysis with anti-His-tag antibody also confirmed the expression of the His-tagged protein (***Fig. 1***). The protein was purified by Ni-sepharose affinity chromatography in denaturing condition and eluted at pH 4.5. After refolding, the purified iRpfE migrated at a molecular weight of 22.0 kDa on 15% SDS-PAGE (***Fig. 2***). Finally, about 1.65 mg of the purified iRpfE proteins were obtained from 200 mL of culture.

### Expression and purification of recombinant sRpfE in *M. vaccae*

**Fig. 3:**
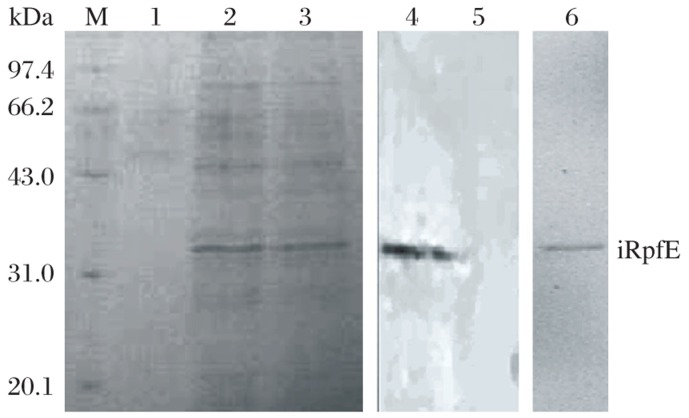
Expression and purification of sRpfE on 15% SDS-PAGE gel and Western blot analysis using anti-His6 antibody. Lane M: molecular weight marker; Lanes 1-3: elution with native elution buffer; Lane 4: Western blot analysis of lysate of *M. vaccae* (pDE22-rpf) using His antibody; Lane 5: Western blotting analysis of lysate of *M. vaccae* (pDE22) using His antibody; Lane 6: purified sRpfE.

The plasmid pDE22-rpfE was electroporated into the competent *M. vaccae*. The expressed sRpfE was purified with Ni-Sepharose. The purified protein was also present at a molecular weight of 22.0 kDa on 15% SDS-PAGE (***Fig. 3***). Western blot analysis with anti-His-tag antibody was performed to validate the expression. Finally, 0.26 mg of the sRpfE protein was obtained from 200 mL of culture.

### Antibody response to iRpfE and sRpfE

IgG_1_ and IgG_2a_ were detected from mice immunized with iRpfE and sRpfE. Both RpfE proteins elicited high levels of IgG_1_ against RpfE (***Table 1***). The results were depicted at a serum dilution of 1:500, since 1:100 dilution provided ODs corresponding to the saturation plateau of the titration curve created by specific antibodies to both RpfE proteins. In addition, both proteins also elicited appreciable levels of IgG_2a_ response (***Table 1***). In the BCG group, the levels of IgG_2a_ and IgG_1_ were significantly higher than those in the iRpfE and sRpfE groups (*P* < 0.001).

**Table 1:** Levels of antibodies to the RpfE proteins in sera

Immunogen	IgG subclass (OD_420_)^a^	
	IgGl (1:500)	IgG2a (1:100)
iRpffi	1.042 ± 0.027	0.394 ± 0.057
sRpffi	1.428 ± 0.032	0.367 ± 0.041
BCG	1.623 ± 0.014	0.764 ± 0.036
PBS	0.089 ± 0.012	0.061 ± 0.017

^a^Sera from five animals in each group were evaluated individually at the dilutions indicated. Results are described as mean ± SD of OD_420_ values for each group. The data shown is a representative of two repeated experiments.

### T cell proliferation

**Fig. 4:**
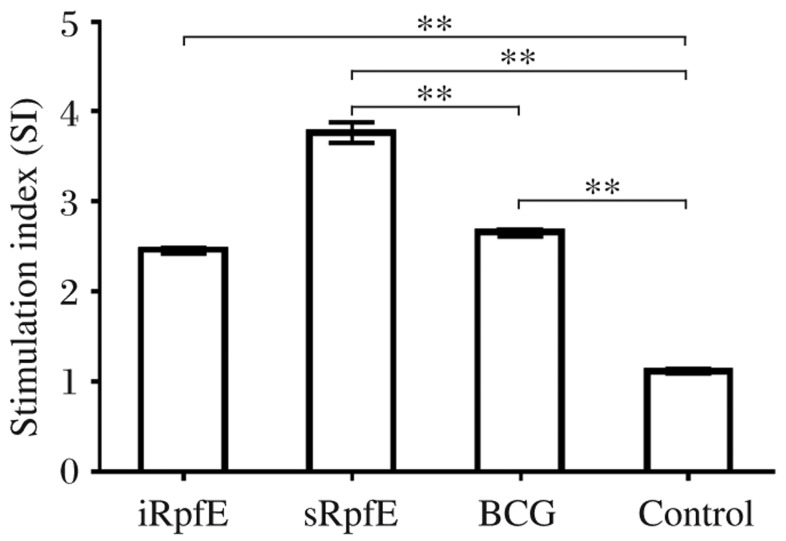
Proliferation of splenocytes induced by RpfE fusion proteins and BCG. Splenocytes from mice injected with PBS and incomplete Freund's adjuvant (IFA) were used as negative controls. The stimulation index (SI) was calculated by the OD_490_ values of the experimental group divided by those of the controls. The results are expressed as mean±SD, and all experiments were repeated three times, ^**^P < 0.001.

To evaluate the cell-mediated immune response, the stimulation index (SI) of the splenocytes in immunized mice was measured by the MTT method. The SI values of iRpfE, sRpfE and BCG groups were (2.46±0.08), (3.76±0.25), and (2.65±0.09), respectively, whereas they were (1.11±0.07) in the control group. The SI values were significantly higher in the immunization groups than those in the control group (*P* < 0.001) (***Fig. 4***). In addition, a significantly higher SI was observed in the sRpfE group compared with that of the BCG group (*P* < 0.001), while the SI value in the iRpfE group was similar to that in the BCG group.

### Cytokine production

**Fig. 5:**
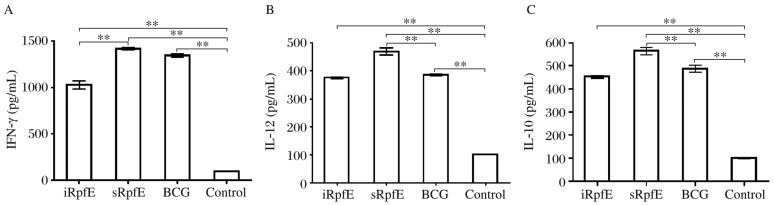
Levels of IFN-γ (A), IL-12 (B) and IL-10 (C) induced in the culture supernatant of splenocytes from mice immunized with purified Rpf proteins and BCG. The culture supernatants of splenocytes from mice injected with PBS and incomplete Freund's adjuvant (IFA) were used as negative controls. The results are expressed as mean±SD, and all experiments were repeated three times, **P < 0.001.

The levels of IFN-γ secretion stimulated by specific antigen were detected by indirect ELISA, and were (1,420±34), (1,030±90) and (1,350±49) pg/mL, respectively, in the cultured supernatants of the splenocytes from the mice immunized with sRpfE, iRpfE and BCG, whereas (99±4) pg/mL (***Fig. 5A***) was detected in the PBS group. The IFN-γ levels were significantly higher in the immunized groups than those in the control group (*P* < 0.001). In addition, the IFN-γ level in the sRpfE group was similar to that in the BCG group (*P* = 0.032), while a higher level was detected in the iRpfE group compared with the BCG group (*P* < 0.001).

The levels of IL-12 and IL-10 secretion stimulated by specific antigens were also detected by indirect ELISA. The IL-12 levels were (469±27), (376±12) and (386±12) pg/mL, respectively, in the cultured supernatants of the splenocytes from the mice immunized with sRpfE, iRpfE and BCG (***Fig. 5B***), whereas the level was (102±6) pg/mL in the control group. A significantly higher IL-12 level was detected in the sRpfE group than that in the BCG group (*P* < 0.001), while the level in the iRpfE group was similar to that in the BCG group (*P* = 0.22). The IL-10 levels were (565±35), (452±15) and (487±23) pg/mL, respectively, in the mice immunized with sRpfE, iRpfE and BCG (***Fig. 5C***), whereas the levels were (99±8) pg/mL in the control group (***Fig. 5C***). The production of IL-10 was significantly higher in the sRpfE group than that in the BCG group (*P* = 0.007), while similar level was produced in the iRpfE and BCG groups (*P* = 0.064). Generally, the sRpfE-stimulated cells produced larger amounts of IL-12, IL-10 and IFN-γ in an antigen-specific manner than those stimulated by iRpfE, which is consistent with the results of a previous report using other Rpf proteins. sRpfE appeared to be the best inducer of type 1 cytokine response as stimulated by *M. tuberculosis*.

## DISCUSSION

Nearly all expressed proteins are found in the inclusion bodies. Inclusion bodies can potentially be a good starting point for the purification of proteins, since they contain almost pure proteins in different states of aggregation in an inactive form. However, the main problem lies in the correct refolding of fully active protein.

The shuttle vector pDE22 is derived from a vector pSMT3, and also contains the pAL5000 origin of replication, the gene for hygromycin resistance, the HSP60 promoter and has the signal sequence from the BCG alpha gene. This vector can also be used as expression vector in *M. vaccae*[20]. *M. vaccae* is a fast-growing mycobacterial species, and is homologous to *M. tuberculosis*, indicating that the recombinant sRpfE expressed in *M. vaccae* may be similar to the native RpfE in *M. tuberculosis*. The proteins expressed by pDE22 are secreted into the culture supernatant. Considering that pDE22 contains no tags for purification, we therefore designed a sequence encoding a His-tag on the *rpfE* gene to facilitate the purification.

Secreted and surface-exposed cell wall proteins are major antigens recognized by the protective immune response against *M. tuberculosis*. Immunization with whole-culture filtrate, a rich source of these extracellular proteins, can protect mice and guinea pigs to some extent against subsequent challenge with the tubercle bacilli[21]. Since RpfE is one of the secreted proteins, we also assessed the cytokine production by splenocytes in mice immunized with RpfE proteins. IFN-γ has been well established as a protective cytokine in animal models of TB[22]. IL-12 is essential to the generation of a protective immune response to *M. tuberculosis.* Its main functions include induction of IFN-γ expression and the activation of antigen-specific lymphocytes capable of creating a protective granuloma[23],[24]. Mycobacteria and other intracellular pathogens are potential inducers of IL-10, and diseases caused by these organisms are frequently associated with the immunologic unresponsiveness and failure to produce IFN-γ[25],[26].

In summary, the present study showed that RpfE protein expressed in *E. coli* and *M. vaccae* elicited cellular immune response in immunized mice. RpfE purified from *M. vaccae* exhibited better efficiency than BCG in the production of IL-10 and IL-12. The challenge of immunized mice with *M. tuberculosis* will be further elucidated to investigate the potential of this protein as subunit vaccine.
